# High current density GaAs/Si rectifying heterojunction by defect free Epitaxial Lateral overgrowth on Tunnel Oxide from nano-seed

**DOI:** 10.1038/srep25328

**Published:** 2016-05-04

**Authors:** Charles Renard, Timothée Molière, Nikolay Cherkashin, José Alvarez, Laetitia Vincent, Alexandre Jaffré, Géraldine Hallais, James Patrick Connolly, Denis Mencaraglia, Daniel Bouchier

**Affiliations:** 1 IEF, CNRS, Univ Paris-Sud, Université Paris-Saclay, Orsay, France; 2 GeePs, UMR CNRS 8507, CentraleSupelec, Univ Paris-Sud, Sorbonne Universités, UPMC Univ Paris 06, Université Paris-Saclay, 11 rue Joliot Curie, Plateau de Moulon, 91192, Gif sur Yvette, France; 3 CEMES-CNRS and Université de Toulouse, 29 rue Jeanne Marvig, 31055 Toulouse, France; 4 Universidad Politécnica de Valencia, NTC, Camino de Vera s/n. 46022, Valencia, Spain

## Abstract

Interest in the heteroepitaxy of GaAs on Si has never failed in the last years due to the potential for monolithic integration of GaAs-based devices with Si integrated circuits. But in spite of this effort, devices fabricated from them still use homo-epitaxy only. Here we present an epitaxial technique based on the epitaxial lateral overgrowth of micrometer scale GaAs crystals on a thin SiO_2_ layer from nanoscale Si seeds. This method permits the integration of high quality and defect-free crystalline GaAs on Si substrate and provides active GaAs/Si heterojunctions with efficient carrier transport through the thin SiO_2_ layer. The nucleation from small width openings avoids the emission of misfit dislocations and the formation of antiphase domains. With this method, we have experimentally demonstrated for the first time a monolithically integrated GaAs/Si diode with high current densities of 10 kA.cm^−2^ for a forward bias of 3.7 V. This epitaxial technique paves the way to hybrid III–V/Si devices that are free from lattice-matching restrictions, and where silicon not only behaves as a substrate but also as an active medium.

Alternative GaAs-on-Si substrates have a considerable market potential for replacing the costly GaAs substrate in producing traditional GaAs-based devices such as solar cells, photodetectors, LEDS, lasers, and microwave devices, and as a new technology for monolithic integration of GaAs elements and Si integrated circuits^1^,^2^,^3^,^4^,^5^,^6^,^7^. The first step toward this goal is to obtain high quality GaAs layer on a Si substrate, creating so-called virtual substrates. However, three major problems remain unresolved in GaAs layers grown directly on plain silicon substrates, i) the high density of threading dislocations due to the lattice mismatch with Si (around 4%), ii) the formation of anti-phase domains (APDs) due to the polar/non-polar semiconductor interface and iii) the formation of cracks due to the difference in thermal expansion coefficients of GaAs and Si^8^. Significant improvements have been reported for many years, thanks to selective area epitaxy (SAE) of GaAs on Si substrates patterned with dielectric films^9^,^10^,^11^,^12^. Moreover, even if these deposition procedures can keep the greater part of the epilayer free of defects, the relaxation process of GaAs on silicon leads to the presence of a high density of misfit dislocations located at the interface between Si and GaAs. This renders these layers inappropriate for applications involving carrier transport through the interfacial region. A promising technique which overcomes these problems is wafer bonding, which is a non-epitaxial method for III–V thin film integration on Si substrates and therefore not subject to the lattice matching limitations associated with epitaxial growth^13^. Thus, recently Tanabe *et al.* have obtained promising results with direct fusion bonding of GaAs on Si by successfully obtaining ohmic GaAs/Si highly conductive heterojunction through a 2 nm thick amorphous layer at the GaAs/Si interface^5^. Nevertheless, such artificial GaAs/Si wafer organization is limited in size by the available III–V substrates, and as the donor GaAs wafer must be eliminated by etching, this technology is considerably more expensive than the epitaxial route^14^.

Therefore, the direct epitaxial growth of III–V compounds on Si substrates remains the most desirable approach for III–V/Si hybrid integration. We demonstrate in this paper that the epitaxial lateral overgrowth of GaAs on nano patterned Si substrates with dielectric films appears to be the most promising technique. Thus, if this technique is coupled with the use of a sufficiently small nucleation area size, it is expected to enable the relaxation of the mismatched material without emission of misfit dislocations^15^. The other advantage of starting from small nucleation areas is to avoid the formation of steps inside nanoseeds, and as the Si surface is monodomain, the growing GaAs crystal cannot form APDs. We have previously demonstrated APDs and dislocation-free GaAs regions grown on thin SiO_2_ layer where the crystalline order between epi-GaAs and Si substrate was transferred through nano-holes in the SiO_2_ layer^16^,^17^. This first demonstration was done on (001) substrates. In the present paper, we show that some defects remaining on (001) are avoided on (111) orientation. Moreover, a tunneling GaAs/Si heterojunction through the thin SiO_2_ layer can be expected. We propose to call this growth method ELTOn for Epitaxial Lateral overgrowth on Tunnel Oxide from nano-seed. This integration process presents a promising route to ultrahigh efficiency III–V/Si tandem solar cells with a close to ideal bandgap combination^18^ which are free from lattice-matching constraints.

## Results

### GaAs/Si epitaxial growth

**Figure 1:**
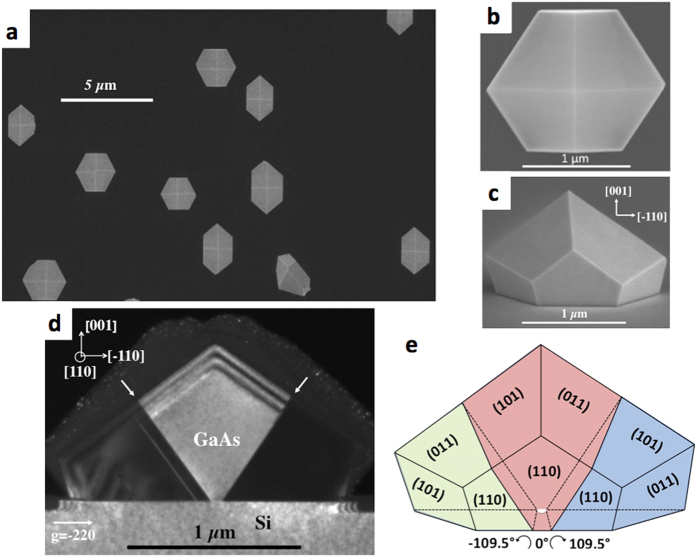
GaAs/Si epitaxial growth on (001)Si. Plan view of a scanning electron microscopy (SEM) image of (**a**) several microscale GaAs crystallites, and (**b**) zoom of one microscale GaAs crystallite, grown on a (001)Si surface through seeds within a thin SiO_2_ layer. (**c**) tilted view around the [110] axis of a microscale GaAs crystallite. (**d**) Dark-field cross-sectional image of the island taken close to the [110] zone axis of Si with g = −220. The crystal in bright contrast is in perfect epitaxy with the Si(001) substrate. {111} GaAs twin planes between the central and the left/right parts of the GaAs island are marked by arrows. (**e**) Schematic representation of the structure of the half of a GaAs island with the identified planes of the facets and twin planes.

Isolated GaAs microcrystals were grown on (001)Si and (111)Si substrates by the method of Epitaxial Lateral overgrowth on Tunnel Oxide from nano-seed (ELTOn) described in the Methods section of this paper. Their morphology was investigated by transmission electron microscopy (TEM) and their local electrical characterizations were performed by conductive-probe atomic force microscopy (CP-AFM) and Electron Beam Induced Current (EBIC) measurements. Figure 1(a) shows a plane view scanning electron microscopy (SEM) image of several micrometer scale GaAs crystals obtained by ELTOn on (001)Si substrate. Figure 1(b,c) show (001) plane and tilted view SEM images of individual GaAs crystals. The vast majority of GaAs crystals present identical shapes and have 10 {110} facets with a twofold symmetry. This {110}-type orientation is energetically stable and its dominance in faceting can be understood in the nanoscale regime^19^. Figure 1(d) shows cross-sectional (cs) (110) dark-field image of the GaAs micro-crystals taken close to the zone axis [110] of Si with the diffraction vector g = −220. According to the diffraction patterns (DPs) taken at different parts of GaAs micro-crystal grown and in the (001)Si substrate (not shown) we have observed that the micrometer scale GaAs crystal is composed of three differently oriented parts, nominally left, central, and right parts. The central part has the direct interface with the Si substrate and is nearly aligned with the Si substrate. The left and the right parts of the islands lying on the surface of the SiO_2_ layer have twin like orientations with respect to the central part and are clockwise and anticlockwise rotated around the [110] axis by 109.47° with respect to the orientation of the central part. The identification of the GaAs crystal orientation, of the twin and facet planes and the measurement of angles between GaAs crystal edges allows unambiguous reconstruction of the structure of the GaAs island. For the sake of clarity a schematic representation of the structure of half a GaAs μ-crystal (the omitted part has the same mirror symmetry) is shown in Fig. 1(e). In a previous paper^16^, a simple model was proposed to explain the twin pair formation. One can expect that the twin formation proceeds as soon as the growing crystal reaches the Si to SiO_2_ boundary, which locally alters the crystalline order. In the [110] direction the growing GaAs crystal can wet the SiO_2_ layer without perturbation regarding the chemical bond orientations. This also means that lateral growth can spread along the [110] direction to form a vertical (110) external facet. On the contrary, in the [−110] direction, the As atoms at the edge of the SiO_2_ layer cannot bind with a fourth bottom Ga atom, and thus exhibit a dangling bond. This frustration would provoke the observed anticlockwise rotation of the GaAs lattice by 109.47° around the [110] direction.

Complementary TEM experiments have also been performed in order to determine the presence or absence of antiphase domains, and as the contrast was found to be homogeneous and unchanged when changing Bragg vectors (from g = 002 to g = 00 − 2), we can deduce that no APDs were present^16^. Some statistical SEM and TEM analysis of the micrometer scale GaAs/Si(001) crystals exhibiting regular shapes evidence that nucleation from nanoscale openings avoids the formation of APDs and misfit dislocations. Nevertheless, as we have shown, this GaAs on (001)Si growth technique necessarily leads to the creation of twin pairs. This is due to the polar nature of GaAs materials and to the related asymetry between the directions^1^,^2^,^3^,^4^,^5^,^6^,^7^,^8^,^9^,^10^ and [110] which perturbates the ELTOn growth on (001)Si substrate. One way to overcome this problem is to achieve growth on a surface free of this kind of asymetry, such as the (111) surface.

**Figure 2:**
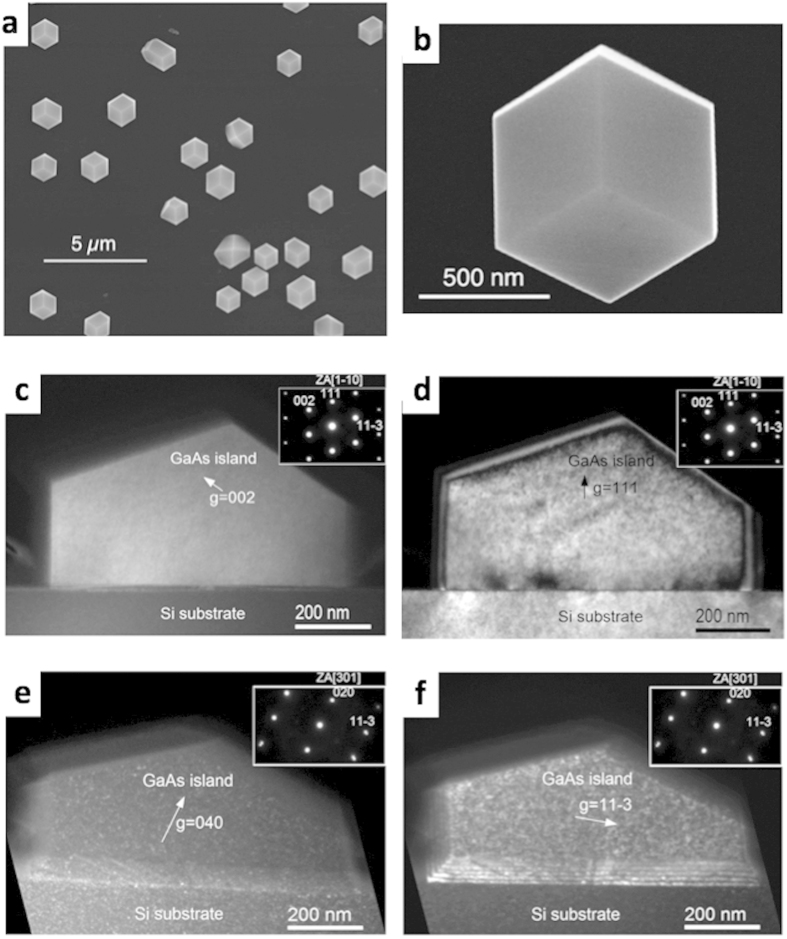
GaAs/Si epitaxial growth on (111)Si. Plan-view of a scanning electron microscopy (SEM) image of: (**a**) several microscale GaAs crystallites and (**b**) zoom of one microscale GaAs crystallite, grown on a (111)Si surface through the seeds within a thin SiO_2_ layer. (**c–f**) Dark-field cross-sectional (1–10) TEM images of the ELTOn GaAs/(111)Si micro-crystal obtained at two-beam conditions: (**c**)^1^,^2^,^3^,^4^,^5^,^6^,^7^,^8^,^9^,^10^ zone axis, g = 002, exact Bragg conditions; (**d**)^1^,^2^,^3^,^4^,^5^,^6^,^7^,^8^,^9^,^10^ zone axis, g = 111, exact Bragg conditions; (**e**) [301] zone axis, g = 040, (g, +2g) weak-beam conditions; (**f**) zone axis [301], g = 11–3, (g, +2g) weak-beam conditions. (**e**) and (**f**) were obtained by a ∼48° rotation around the^3^,^4^,^5^,^6^,^7^,^8^,^9^,^10^,^11^ axis from the^1^,^2^,^3^,^4^,^5^,^6^,^7^,^8^,^9^,^10^ zone axis.

Therefore, the GaAs ELTOn process was also performed on (111)Si wafers. Figure 2(a) shows a plane view SEM image of several micrometer scale GaAs crystal obtained by ELTOn on a (111)Si substrate, and Fig. 2(b) shows a plane view SEM image of an individual GaAs crystal of hexagonal shape with 9 {110} facets. This indicates that the equilibrium shape of GaAs crystal on Si is achieved by exhibiting {011} facets that are non-polar surfaces and have a lower surface energy than other facets. Figure 2(c,d) shows the dark-field cross-sectional (1–10) TEM images of the ELTOn GaAs/(111)Si micro-crystal obtained close to the^1^,^2^,^3^,^4^,^5^,^6^,^7^,^8^,^9^,^10^ zone axis at two-beam, exact Bragg conditions with g = 002 (Fig. 2(c)) and g = 111 (Fig. 2(d)). The micrometer scale GaAs crystal is nearly aligned with the Si substrate. From these images we can deduce that neither twins nor APD were formed in the GaAs micro-crystal. Figure 2 (e,f) shows the dark-field weak-beam images of the same crystal obtained by a ≈48° rotation around the^3^,^4^,^5^,^6^,^7^,^8^,^9^,^10^,^11^ axis (indicated in inserts in Fig. 2(c–f)) from the^1^,^2^,^3^,^4^,^5^,^6^,^7^,^8^,^9^,^10^ zone axis and taken close to the [301] zone axis with g = 040 (Fig. 2(e)) and g = 11 − 3 (Fig. 2(f)). Such complementary TEM observations allow to visualize the projected tilted Si/SiO_2_/GaAs and Si/GaAs interfaces. By using visibility criteria of dislocations^20^, we can indicate the absence of dislocations neither at the interfaces nor within the whole volume of the GaAs micro-crystal. A statistical SEM and TEM analysis concerning micrometer scale GaAs/Si(111) crystals of regular shape enabled to confirm the absence of extended defects within such crystals. Therefore, following this geometry the ELTOn of GaAs leads to a perfect integration of GaAs on Si. It should be noted that the growth temperatures used here are lower than 575 °C. This demonstrates the compatibility of our growth process with the Si CMOS technology platform, as temperature should be preferably limited to a maximum of 600 °C after CMOS processing. Therefore, the epitaxy step can be integrated with the CMOS process without degrading pre-existing MOS device elements. Nevertheless, we have to keep in mind that the CMOS process is limited to 001-oriented substrates. We have now to investigate if this stacking is appropriate for applications involving electronic transport through the interfacial region.

### Local electrical characterizations of GaAs/Si microscale islands

It should be noted that the SiO_2_ layer between the GaAs/Si interface has a thickness of around 0.6 nm for (001)Si, and of 1.2 nm for (111)Si. Tanabe *et al.* have previously shown that GaAs directly bonded on Si substrate through an amorphous layer up to 2 nm thick can lead to a highly conductive heterojunction^5^. Therefore, the SiO_2_ thicknesses used for the present study are sufficiently thin to provide high interfacial conductivity via tunneling^21^,^22^.

To check this point, local electrical characterizations of GaAs/Si microscale islands were performed by CP-AFM^23^,^24^,^25^,^26^. In addition to CP-AFM, EBIC has also been applied to electrically map the GaAs/Si cross-section.

**Figure 3:**
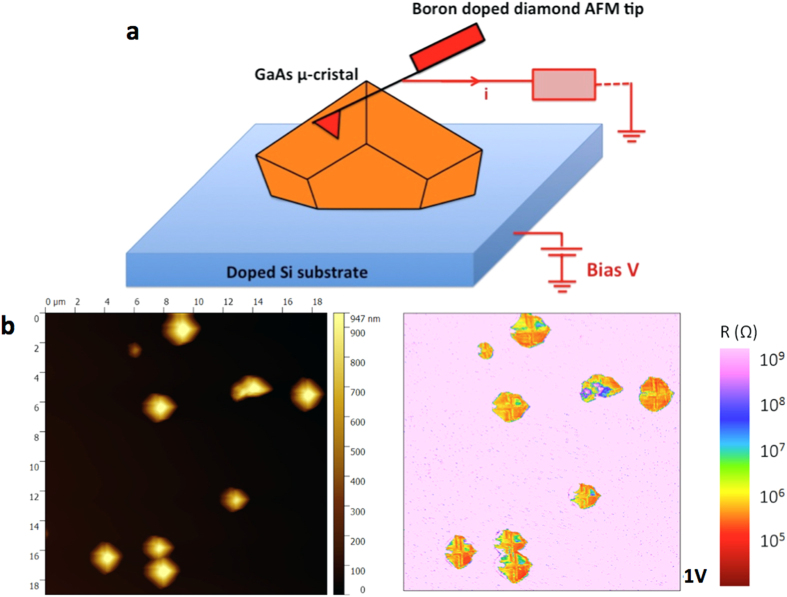
Conductive probe–AFM measurements. (**a**) Sketch illustrating the details of CP-AFM measurements on GaAs μ-cristal obtained by ELTOn process. (**b**) 20 × 20 μm^2^ surface map illustrating the topography (left side) and the local resistance (right side) of GaAs μ-cristal obtained by ELTOn process on (001) p-Si wafers ([B] = 3.3–4.5 × 10^15 ^cm^−3^).

**Figure 4:**
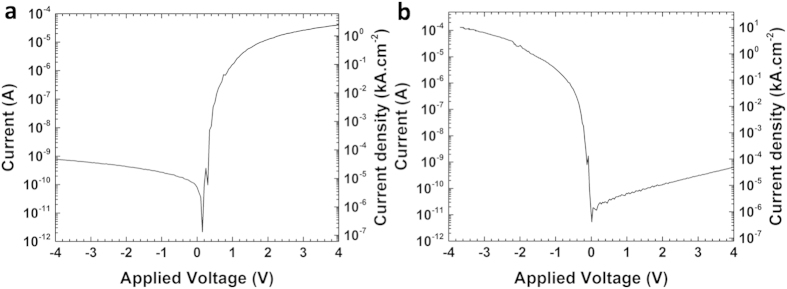
I–V measurements. I–V characteristics, measured by CP-AFM, of the ELTOn GaAs/Si μ-crystal on (**a**) (001) p(B) Si and (**b**) (001) n(P) Si.

**Figure 5:**
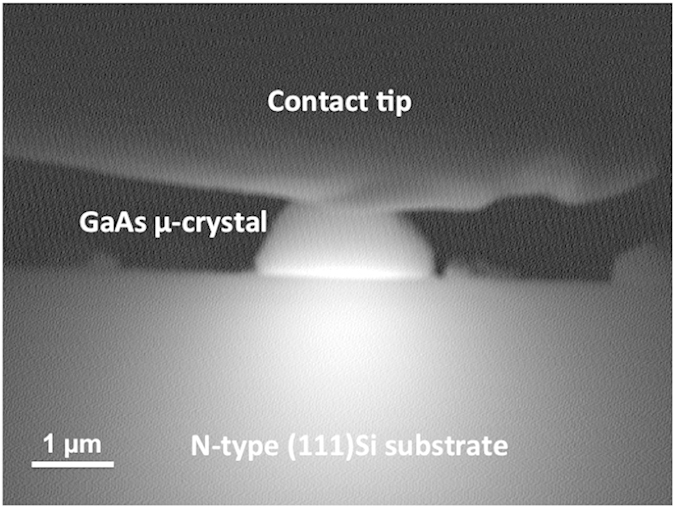
Electron beam induced current map. EBIC map superimposed with the SEM structural image of ELTOn GaAs microcrystal grown on (111) n-Si wafers ([P] = 1.1–8.3 × 10^16^ cm^−3^).

AFM measurements were performed simultaneously in the topographical mode and in the conductive-probe mode (Resiscope™). By applying a voltage bias between the substrate and the conducting cantilever, a current is generated. This current can be used to construct a spatially resolved conductivity image. It also allows for local current vs voltage measurements (I–V) with purely topographic feedback and high resolution. These measurements were very useful to investigate the local electrical behaviour of GaAs/Si-substrate heterojunctions. The CP-AFM details and more specifically the sample configuration and biasing are displayed in Fig. 3a. The DC bias voltage was applied through the doped silicon wafer. Figure 3b depicts a 20 × 20 μm^2^ surface map that illustrates, from left to right, the topography and the electrical properties of n.i.d (non-intentionaly doped) GaAs μ-crystals obtained by ELTOn process. The brightest spots (highest features) in the topography image represent the GaAs μ-crystals that are well correlated with the conductive yellow-green spots in the electrical image obtained for an applied voltage of +1V. This last image results from an average of the electrical images scanned in trace and retrace directions to minimize the closed loop faults that can induce the topography of the μ-crystals. In order to get more precise information about the variation of the local resistance as a function of the applied bias, CP-AFM was locally used to investigate I–V characteristics on individual GaAs μ-crystals grown on Si(001) and Si(111). Figure 4 displays a semi-log plot of the I–V characteristics, from left to right, of ELTOn GaAs μ-cristals grown on (001) p(B)-Si wafer (3.3 × 10^15^–4.5 × 10^15^ cm^−3^) and on (111) n(P)-Si wafer (1.1 × 10^16^ −8.3 × 10^16^ cm^−3^). Figure 4a,b revealed rectifying properties and non-ohmic behavior (diode-like) with currents as high as 1.3 × 10^−4 ^A and 4.2 × 10^−5 ^A under forward bias, respectively, for n-doped (111)Si and p-doped (001)Si. These rectifying behaviours were observed for all the analyzed μ-crystals and evidenced for different AFM tips in terms of electrical coating (platinum silicide and Pt-Ir). Statistically, all the GaAs show a rectifying behaviour, and identical forward and reverse currents were also observed for GaAs μ-cristals presenting similar shape and size. Nevertheless, some differences were observed for the reverse current pointing out multiple origins (μ-crystal and tunnel-oxide areas, quality of electrical contact between the AFM tip and the μ-crystal, etc) that are still under investigation. In order to further characterize the GaAs/Si-substrate heterointerfacial electrical properties, complementary EBIC measurement was also performed on ELTOn GaAs micro-crystals on Si wafers. Two contacts were taken with nano-tips on the sample, the top one on the GaAs micro-crystal and the lower one on the side of the Si wafer. As expected, the p-n heterojunction between GaAs micro-crystal and Si substrate exhibits an internal electric field (which induces to the observed EBIC current by separating e-beam generated electron–hole pairs). This is shown in Fig. 5 displaying the EBIC contrast around the GaAs and Si interface superimposed with the SEM structural image of a GaAs micro-crystal grown on (111) n-Si. The EBIC map indicates that the current crosses uniformly through the whole interface between Si subtrate and ELTOn GaAs micro-crystal including the germination hole and the oxide area. This confirms that the GaAs/Si heterojunction through the thin SiO_2_ layer is conductive. Moreover, I–V characteristics obtained from EBIC are comparable to those obtained by CP-AFM. As the current crosses uniformly through the GaAs/Si interface, we can deduce current densities from the contact area between GaAs μ-crystal and SiO_2_ layer. Current densities as high as 10 kA.cm^−2^ and 2.5 kA.cm^−2^, respectively for n-doped (111)Si and p-doped (001)Si, have been measured in forward bias. These current densities values are much higher than those obtained by direct GaAs/Si bonding technique^5^,^27^ and are comparable with those obtained for Si-InAs heterojunction fabricated by growing InAs nanowires in oxide mask openings on silicon^28^,^29^.

### GaAs/Si heterojunction modelling

In order to determine the (non intentional) doping level of our GaAs micro-crystal, calculations of I–V characteristics of the GaAs/Si heterojunction with varying doping concentrations of GaAs have been performed, and compared to experimental I–V characteristics. The SILVACO modelling^30^ uses numerical solutions extensively described in the literature and well adapted to this system. The aim of this numerical modelling is to establish the likely polarity and density of net doping in the GaAs crystals, and to qualitatively reproduce the trends observed under forward and reverse bias. The junction area is estimated from the micrographs and is set at 1 μm^2^ in the simulations, and the height of the GaAs crystal is set to 1 μm. The modelling is insensitive to the thickness of the Si region, which is therefore set to 10 μm for computational reasons rather than the experimental thickness of 200 μm.

The modelling assumes that the GaAs doping level must be the same for ELTOn GaAs μ-cristals grown on (001) p(B)-Si wafers and on (111) n(P)-Si wafers. A good agreement is found for a p-type doping in the 10^15^–10^16^ cm^−3^ range in ELTOn GaAs μ-crystal. This low p-type doping level is in agreement with the value already reported by Kikkawa *et al.*, for growth conditions pretty close to ours, in terms of temperature and V/III ratio with the use of TMGa and TBAs precursors^31^.

## Conclusion

In conclusion, we have proposed a technique to grow epitaxially high quality microscale GaAs crystals on Si subtrate. This method is based on the GaAs chemical beam lateral epitaxy from nanoscale Si seeds opened through a very thin SiO_2_ layer. As a tunnel junction is expected between the GaAs micro crystal and the Si, we have proposed to call this technique ELTOn for Epitaxial Lateral overgrowth on Tunnel Oxide from nano-seed. Transmission electron microscopy have revealed that ELTOn GaAs microcristals grown on (111)Si are perfectly integrated and defect free. Those grown on (001)Si contain only a pair of {111} twin planes sectioning their volume into a central part quasi-similarly oriented with the Si substrate and into two mirror twinned ones. We have experimentally shown that ELTOn GaAs micro-crystals form highly conductive rectifying junctions with the Si substrate whatever its orientation. We have then demonstrated for the first time a monolithically integrated GaAs/Si diode with a current as high as 10 kA.cm^−2^. Finally, a doping level of 10^15^–10^16 ^cm^−3^ range in GaAs micro-scale islands was found to account for the I–V characteristics measured with complementarily doped Si substrates.

Therefore, in comparison with other typical hetero-epitaxial methods of III–V growth on Si, the ELTOn method appears as very promising for monolithic integration of III–V semiconductor on Si, where Si not only behaves as a substrate but also as an active medium. For the purpose of developing electronic devices, the development of localized nano-seeds, as well as post-epi planarization process is now under development.

## Methods

### Epitaxial growth

The Epitaxial Lateral overgrowth on Tunnel Oxide from nano-seed (ELTOn) technique proposed here was carried out in a chemical beam epitaxy system with a base pressure of 2 × 10^−8 ^Pa. Pure silane (SiH_4_), trimethylgallium (TMGa) and tertiarybutylarsine (TBAs) were used as gas sources. The (001) p(B)-Si wafers (3.3 × 10^15^–4.5 × 10^15 ^cm^−3^) and (111) n(P)-Si wafers (1.1 × 10^16^–8.3 × 10^16^ cm^−3^) were cleaned using the modified Shiraki chemical cleaning^32^ which results in a reproducible 0.6 nm for (001)Si, resp. 1.2 nm for (111)Si, thick oxide layer (deduced from Auger electron spectrometry and HRTEM). After this chemical cleaning step, the substrates were slowly annealed *in situ* up to 650 °C, the pressure being maintained below 3 × 10^−6 ^Pa.

Prior to GaAs epitaxy, the nucleation seeds must be created. In order to open nanoscale holes through the thin silica layer, the oxidized substrate was exposed to a silane partial pressure of 0.66 Pa at a temperature less than 650 °C during 4 min for Si(001) and 1 min 30 s for Si(111). That results in pure Si nano-areas formed via the reduction of SiO_2_ into volatile SiO. This method which has been detailed elsewhere^33^ has the advantage of easily providing nanoscale seeds around 50 nm in width, randomly spaced at the surface of the oxidized Si substrate. After this step, the GaAs epitaxy was initiated with the well-known two step procedure^8^. The first step consists in introducing solely TBAs (during 2 min) under a total pressure of 1.32 Pa at 430 °C. In the second step TMGa was also introduced and the total pressure was increased up to 6.6 Pa achieving a V/III ratio of [TBAs]/[TMGa] = 6, while the growth temperature was increased up to 550 °C for (001)Si and 575 °C for (111)Si, respectively. After this sequence that tooks 2 min, GaAs epitaxial lateral overgrowth was then continued for 40 min.

### Structural characterisation

The surface morphology of micrometer scale GaAs crystals was characterized by scanning electron microscopy (SEM) (Hitachi SU8000) and the structural and crystalline quality of micrometer scale GaAs were further investigated by cross-sectional transmission electron microscopy (TEM) using a Jeol 2010 TEM operating at 200 kV, from lamellas prepared by gallium focused ion beam (Ga-FIB) milling.

### Conductive-probe AFM measurements

Local electrical measurements of GaAs/SiO_2_/Si micro-structures were investigated with conductive-probe AFM (CP-AFM) and were performed using a Digital Instruments Nanoscope IIIa Multimode AFM associated with the home-made conducting probe extension called “Resiscope™”^23^. This setup allows us to apply a stable DC bias voltage (from −10 to +10 V by 0.01 V steps) to the device and to measure the resulting current flowing through the tip as the sample surface is scanned in contact mode. Local resistance values can be measured in the range of 10^2^–10^12^ Ω, which allows investigations on a variety of materials^34^,^35^ and devices^25^,^36^. Measurement accuracy based on calibrations is below 3% in the range of 10^2^–10^11^ Ω, and it can reach 10% for higher resistance values. In this paper, a boron-doped diamond AFM tip has been used for the local current vs voltage measurements.

### Electron beam induced current measurements

Electron beam induced current (EBIC) measurements have been performed on GaAs/Si heterojunctions in a Hitachi SU8000 SEM equipped with a Gatan EBIC system (SmartEBIC) and *in-situ* nanomanipulators for the electric conctact. This technique employs an electron beam to induce a current within the sample which is used as a signal for generating images that depict the electrical behaviour of the sample. With proper electrical contacts the movement of the holes and electrons generated by the SEM’s electron beam can be collected, amplified, analyzed, and displayed as variations of contrast in an EBIC image. Samples were polished on the edge. Two tungsten micro-tips supported by micromanipulators enable in the SEM, to contact the upper top of a GaAs cristal and the backside of the Si substrate, and to collect I(V) curves or induced current. The electron beam is scanned perpendicular to the cross section i.e. perpendicular to the normal of the substrate. The electron beam acceleration voltage was set to 25 keV and extraction at 5 μA giving a current on the sample around 150 pA.

### GaAs/Si Modeling

SILVACO modeling^30^ was also used in order to qualitatively reproduce the trends observed for experimental I(V) under forward and reverse bias. Materials parameters used in the simulations use default values in the Silvaco ATLAS manual, appendix B “Materials Parameters” for 300 K standard test conditions. Important GaAs values for this problem are the bandgap of Eg(GaAs) = 1.42 eV, electron affinity χ(GaAs) = 4.07 eV, and relative permittivity ε(GaAs) = 13.2. The equivalent values for Si are Eg(Si) = 1.08 eV, electron affinity χ(GaAs) = 4.05 eV, and relative permittivity ε(GaAs) = 11.8.

## Additional Information

**How to cite this article**: Renard, C. *et al.* High current density GaAs/Si rectifying heterojunction by defect free Epitaxial Lateral overgrowth on Tunnel Oxide from nano-seed. *Sci. Rep.* **6**, 25328; doi: 10.1038/srep25328 (2016).
