# Erratum: High current density GaAs/Si rectifying heterojunction by defect free Epitaxial Lateral overgrowth on Tunnel Oxide from nano-seed

**DOI:** 10.1038/srep29875

**Published:** 2016-09-22

**Authors:** Charles Renard, Timothée Molière, Nikolay Cherkashin, José Alvarez, Laetitia Vincent, Alexandre Jaffré, Géraldine Hallais, James Patrick Connolly, Denis Mencaraglia, Daniel Bouchier

Scientific Reports 6: Article number: 2532810.1038/srep25328; published online: 05 04 2016; updated: 09 22 2016

In the original HTML version of this Article, all instances of the orientation [1–10] and [3–11] were incorrectly written as ^“1,2,3,4,5,6,7,8,9,10”^ and ^“3,4,5,6,7,8,9,10,11”^ respectively. In the original PDF version of this Article, all instances of the orientation [1–10] and [3–11] were given as ^“1–10”^ and ^“3–11”^ respectively. These errors have now been fixed in the HTML and PDF versions of this Article.

